# Disease of the Kidney

**Published:** 1842-12

**Authors:** 


### Selections from American and Foreign Journals.

---

*Disease of the Kidney.*—Dr. Barlow has narrated a series of interesting cases illustrative of the following points:—First, that there is a certain symptom connected with irritation of the kidney, and which, although not confined to it alone, is not necessarily connected with disease of those structures whose affections are most likely to be confounded with those of the kidneys, so that when this symptom is absent, we may eliminate, and thereby disembarrass ourselves of the consideration of all such affections of the kidneys as would necessarily be attended by irritation of that organ; and when this symptom is present, we are furnished with a reason for assigning the seat of the disease to the kidney, in preference to several adjacent structures. The symptom is sickness, or irritability of stomach.

Secondly, and further, there is a certain symptom, or rather, set of symptoms, dependent on the non-depuration of the blood by the kidney, whether this non-depuration be the result of mechanical obstruction to the flow of the urine, of a diminution in its quantity, or of a depraved condition of that secretion, in which its most important ingredients are wanting—viz., cerebral disorder of a peculiar character.

There are, however, several affections of the kidney which do not necessarily give rise to irritation of that organ, and in which the sickness *may* not be present to aid our diagnosis; of these, the principal are the granular degeneration, suppuration, and perhaps some forms of adventitious or malignant deposit.

In the first of these, the disease, depending in the first place probably upon congestion, and afterwards upon a chronic change in the structure of the organ, irritation is not a necessary concomitant, although the disease may, and no doubt frequently does, result from irritation, giving rise to chronic inflammation; accordingly, we find that sickness is not always present, although it is sometimes a most troublesome symptom. In suppuration we should generally have irritation, giving rise to sickness, in the first instance, and probably, at a later period, obstructed function, with its concomitant changes in the urine, and cerebral disorder, with probably, at some time, the presence of pus in the urine.

With regard to malignant disease, it is very probable that this deposit taking place, as has been remarked by Dr. Bright, in the cellular membrane connecting the firm parts of various structures, the parts of the organ in which this deposit takes place may be, as it were, pushed aside, without suffering any mechanical violence or functional disturbance; at the same time, it is also probable that, in the progress of distension to which this organ is subjected, some slight laceration, or irregular pressure, giving rise to irritation, will sooner or later occur, and accordingly some sudden invasion of sickness almost uniformly happens before such deposit in the kidney has produced a tumor of any considerable size. We should also in this case, probably, have some degree of hæmaturia, especially if the disease be of a fungoid character, the most common form of malignant deposit in the kidney.

Irritability of the stomach is not to be regarded as pathognomonic of disease of the kidney: but in cases where doubts arise as to whether any disease is to be referred to the kidney, or some neighboring organ, the absence or presence of sickness will go far to decide the question; and further, in cases not unfrequently met with, where the prominent symptom is distressing and obstinate sickness without any assignable cause, especial attention should be directed to the kidneys, and every means used to determine the state of those organs. The same observations will apply very nearly to the second class of symptoms—namely, the cerebral disorder; for as yet we want sufficient evidence to prove that similar derangement of the function of the brain may not be produced by other causes. The cases narrated are nine in number.—*Prov. Med. Journ.*, July 2, 1842.

